# Commentary: Parenting and transdiagnostic youth psychopathology—Reflections on Richmond‐Rakerd et al. (2026)

**DOI:** 10.1002/jcv2.70164

**Published:** 2026-08-23

**Authors:** Christopher J. Trentacosta

**Affiliations:** ^1^ Department of Psychology Wayne State University Detroit Michigan USA; ^2^ Merrill Palmer Skillman Institute Wayne State University Detroit Michigan USA

**Keywords:** early relational health, parenting, population mental health, preventive intervention, transdiagnostic

Parenting characterized by hostility and low levels of warmth is robustly associated with several specific forms of child and adolescent psychopathology such as conduct problems, but there is comparatively less research on parenting and youth psychopathology from a transdiagnostic perspective. Using data from two longitudinal genetically‐informed cohorts, Richmond‐Rakerd et al. (2026)'s contribution to the 2026 Special Issue of JCPP Advances examined links between two broad and widely‐studied dimensions of parenting—hostility/conflict and warmth/involvement—and a shared liability to psychopathology. Both cohorts provided evidence for links between parenting and general “*p*‐factor” psychopathology liability (Caspi et al., 2014), with particularly robust evidence across cohorts of links between hostility/conflict and the *p*‐factor. There was less evidence for links between the parenting dimensions and unique externalizing or internalizing psychopathology liabilities while accounting for the *p*‐factor, although parental hostility in early childhood predicted externalizing problems in late childhood in the Early Growth and Development Study (EGDS) of adoptive families. Key strengths of Richmond‐Rakerd et al.’s study include the use of longitudinal data from adoption and twin studies that account for genetic confounding as well as the analysis of established measures of parenting dimensions and child symptoms of psychopathology, with reports obtained from both mothers and fathers.

Although there were several differences between the EGDS and the Michigan Twin Neurogenetics Study (MTwiNS) cohorts used by Richmond‐Rakerd et al. (2026), the timing of the assessment of parenting was one particularly important distinction. Links between parenting and the *p*‐factor were particularly robust in the EGDS, where parenting was assessed during early childhood. Parenting during the first few years of life is an essential contributor to children's developing competence across several domains and helps prevent emerging psychopathology, with this evidence providing a core foundation for the large and robust literature on early childhood parent‐focused preventive interventions. Therefore, examining reduced risk for transdiagnostic markers of psychopathology within the context of early childhood preventive intervention trials is a fruitful direction for research. In a recent analysis of the multi‐site randomized controlled trial of the early childhood version of the Family Check‐Up (FCU), children from families receiving the FCU had steeper declines in the *p*‐factor across early childhood, which in turn predicted lower levels of the *p* factor in middle childhood and adolescence as well as less polydrug use in adolescence (Tein et al., 2023). Importantly, the early childhood version of the FCU was originally conceived as a program to prevent conduct problems, with these and other findings from the FCU adding to a growing body of literature that positive effects of early preventive intervention programs frequently extend well beyond their intended targets. The tendency of prevention intervention trials (and many non‐intervention longitudinal studies) to focus more narrowly on either externalizing or internalizing problems may reflect long‐standing research disciplinary silos as well as the structure of research funding. The growing evidence linking dimensions of parenting and preventive intervention effects with transdiagnostic indicators, including the *p*‐factor, suggests that reducing disciplinary silos and promoting transdiagnostic funding opportunities could be fruitful, especially if the overarching goal is to reduce the overall burden of child and adolescent mental health.

A cross‐disciplinary perspective that focuses on understanding and reducing transdiagnostic mental health burden fits with the growing emphasis on promoting early relational health as well as the movement toward population mental health. Early relational health has been defined as “a foundational, culturally embedded and developing set of positive, responsive, and reciprocal interactions that… result in emerging confidence, competence, and emotional well‐being for all” (Willis & Eddy, 2022). The emphasis on emotional well‐being aligns with a transdiagnostic perspective on mental health rather than the traditional, narrower emphasis on preventing and treating specific diagnoses or clusters of problems. A population mental health perspective encourages psychological science and practice to transition from a solely individual‐level focus to emphasize population‐level impact, with the creation of a system of universal primary mental health care as one key component of this process (Dodge, 2025). Promoting early relational health, which undoubtedly includes healthy parenting practices, is an essential component of key early life primary mental health care models. In fact, several of the existing programs that Dodge (2025) and others have highlighted to illustrate the potential impact of universal primary mental health care are centered around fostering healthy early caregiving practices. For example, Triple P (Sanders, 1999), a widely studied program that, like the FCU, was historically more narrowly focused on conduct problems, includes a universal level of intervention that broadly disseminates information about positive parenting practices to the community at large via several sources, including the media (e.g., via television commercials). As research accumulates demonstrating that broad dimensions of parenting are associated with children's general *p*‐factor psychopathology, it makes sense to disseminate information about generally healthy parenting practices as widely as possible to all parents of young children, rather than solely directing this broadly applicable parenting information to parents of children who have already been identified as having specific problems.

However, a perspective that places emphasis on disseminating information about broad dimensions of healthy parenting within primary mental health care does not preclude attention to more specific parenting practices that may be linked to unique indicators of psychopathology. Rather, the primary mental health perspective also recognizes the need for specialized care, including specific evidence‐based treatments for specific disorders (Dodge, 2025). Thus, we continue to need research that parses distinct parenting practices and unique intervention strategies to help prevent and treat specific child and adolescent problem behaviors, but with increased attention to how “parenting” is conceptualized so that the conceptualization aligns with the research questions. The conceptualization of “parenting” in the Richmond‐Rakerd et al. (2026) study is illustrative of a strong tendency in longitudinal cohort studies (as well as much of the preventive intervention literature) to measure broad dimensions of parenting such as warmth and conflict using aggregates of several parenting behaviors, each of which are self‐reported by parents on a rating scale. These established measures of broad dimensions of parenting are appropriate when the goal is to examine whether there are links between parenting and transdiagnostic markers such as the *p*‐factor, but they are less suitable when the goal is to elucidate which specific parenting behaviors are linked to unique liabilities for specific indicators of narrower forms of psychopathology.

When the goal is to uncover which specific parenting behaviors are candidates for unique liabilities to narrower forms of psychopathology, it is useful to turn to the literature on clinical populations of children and adolescents and evidence‐based treatments. For example, parental accommodation, defined as parent behaviors intended to reduce child distress (Thompson‐Hollands et al., 2014), plays an important role in the emergence and maintenance of childhood anxiety disorders and is a key component of innovative cognitive‐behavioral treatment approaches. Another example comes from research on a specific aspect of the externalizing dimension of psychopathology, where an adaptation of Parent‐Child Interaction Therapy for children with callous‐unemotional (CU) behaviors focuses on teaching specific parenting behaviors, including expressing high levels of positive emotion and implementing a token economy, that align with the specific difficulties faced by children with CU behaviors (Fleming et al., 2022). The research literature would benefit from longitudinal cohort studies that simultaneously examine broad dimensions of parenting along with these kinds of more specific parenting behaviors. Such studies could parse the extent to which broad dimensions are associated with the general *p*‐factor while specific parenting behaviors predict narrower, unique markers of psychopathology in ways that align with theoretical and conceptual models of specific forms of psychopathology.

Moreover, genetically‐informed cohorts such as the EGDS and MTwiNS could help tease apart the extent to which genetic confounding plays a role in observed associations between specific parenting behaviors and narrow indicators of child and adolescent psychopathology. For example, in an analysis of the EGDS cohort, adoptive mothers' positive reinforcement buffered the association between genetic risk and children's CU behaviors (Hyde et al., 2016). Notably, this analysis used specific coded maternal behaviors from an observational assessment of parenting during a structured task rather than aggregating parents' self‐reports of their own parenting into broader dimensions. An interesting future direction would be to examine whether the link between observed positive reinforcement and CU behaviors holds while accounting for the *p*‐factor as well as the broader parenting dimensions.

In conclusion, Richmond‐Rakerd et al.’s (2026) study of two longitudinal genetically‐informed cohorts adds to a growing body of literature on parenting and transdiagnostic mental health during childhood and adolescence. The focus on links from dimensions of high levels of parental warmth/involvement and low levels of parental hostility/conflict in early childhood to children's subsequent general “*p*‐factor” psychopathology fits with the growing emphasis on the importance of early relational health and has implications for population mental health care. Society will benefit from wider dissemination of evidence‐based information about the broad dimensions of healthy parenting, while researchers and clinicians continue to home in on identifying and addressing specific parenting difficulties that predict and maintain unique markers of more specific forms of psychopathology.

## AUTHOR CONTRIBUTIONS

**Christopher J. Trentacosta**: Writing—original draft; writing—review and editing; conceptualization.

## CONFLICT OF INTEREST STATEMENT

The author declares no conflicts of interest.

## ETHICAL CONSIDERATIONS

Ethics requirements do not apply to this commentary article.

## Data Availability

Data sharing not applicable to this article as no datasets were generated or analyzed during the current study.
